# Analysis and Functional Verification of *PoWRI1* Gene Associated with Oil Accumulation Process in *Paeonia ostii*

**DOI:** 10.3390/ijms22136996

**Published:** 2021-06-29

**Authors:** Jing Sun, Tian Chen, Mi Liu, Daqiu Zhao, Jun Tao

**Affiliations:** 1 College of Horticulture and Plant Protection, Yangzhou University, Yangzhou 225009, China; jingsun@yzu.edu.cn (J.S.); tianchen19970224@163.com (T.C.); MX120180604@yzu.edu.cn (M.L.); dqzhao@yzu.edu.cn (D.Z.); 2 Joint International Research Laboratory of Agriculture and Agri-Product Safety, The Ministry of Education of China, Yangzhou University, Yangzhou 225009, China

**Keywords:** *Paeonia ostii*, *WRINKLED1*, oil synthesis

## Abstract

The plant transcription factor *WRINKLED1* (*WRI1*), a member of *AP2/EREBP*, is involved in the regulation of glycolysis and the expression of genes related to the de novo synthesis of fatty acids in plastids. In this study, the key regulator of seed oil synthesis and accumulation transcription factor gene *PoWRI1* was identified and cloned, having a complete open reading frame of 1269 bp and encoding 422 amino acids. Subcellular localization analysis showed that *PoWRI1* is located at the nucleus. After the expression vector of *PoWRI1* was constructed and transformed into wild-type *Arabidopsis thaliana*, it was found that the overexpression of *PoWRI1* increased the expression level of downstream target genes such as *BCCP2*, *KAS1*, and *PKP-β1*. As a result, the seeds of transgenic plants became larger, the oil content increased significantly, and the unsaturated fatty acid content increased, which provide a scientific theoretical basis for the subsequent use of genetic engineering methods to improve the fatty acid composition and content of plant seeds.

## 1. Introduction

The tree peony, as a famous traditional flower in China, has been cultivated for thousands of years. In addition to its ornamental value, its oil value is also widely recognized. Since being approved as a new resource food by the Ministry of Health of China in 2011 [1], it has been widely promoted by various governments. At present, the tree peony has become the third largest woody oil crop in China after walnut and oil camellia. *Paeonia ostii* is a perennial woody deciduous shrub belonging to the genus Paeoniae in the Paeoniaceae family, which has the characteristics of large flowering, less tillering, high seed yield and strong ecological adaptability. Currently, *P. ostii* is not only the most widely cultivated oil peony variety in China, but also is a vital new oil crop integrating ornamental, medicinal, and oil use [2]. The content of unsaturated fatty acids in *P. ostii* seed oil is rich, among which α-linolenic acid and linoleic acid represent the plant’s advantages over other oil crops because of their higher nutritional value and better economic benefits. To alleviate the contradiction between the production and demand of vegetable oils in China, it is of great significance to improve the oil content and fatty acid composition of woody oil crops by means of biological techniques.

Oil synthesis involves the flow and direction of glycolysis metabolites and the transport coordination of metabolites between different subcellular organelles and cytoplasmic sites. The regulation of plant lipid synthesis is complex, and the research on the lipid metabolism pathway and its structural genes has been relatively clear [3]. In recent years, studies have found that the process of lipid synthesis is strictly regulated at the transcriptional level. At present, some important transcription factors regulating plant lipid metabolism have been found, including five categories: *bZIP*, *B3*, *NFYB*, *ASIL*, and *AP2/EREB* transcription factor *WRI1* [4]. First, *ABI4*, a transcription factor of *bZIP*, can be combined with ABA response elements and can directly combine fatty acids to synthesize the promoter region of catalase *DGAT1* in ABA signaling pathway, thus promoting the synthesis of *DGAT1* [5]. The *GmbZIP123* transcription factor in soybeans can specifically combine the promoter region of *SUC1* and *SUC5* genes to enhance the transport of endogenous sugar, eventually leading to an increase in oil content [6]. Meanwhile, the overexpression of *NsbZIP1* in *Nannochloropsis salina* could significantly enhance the growth and lipid content of transformants [7]. Second, the *B3* transcription factor family plays a vital role in embryo maturation and seed ABA signaling pathway [8]. It has 95, 108, and 81 members in *Arabidopsis*, soybean, and corn [9], respectively, and can specifically identify DNA sequences containing a conservative *RY/Sph* structure. The *B3* transcription factors mainly include *ABSCISIC ACID INSENSITIVE3* (*ABI3*), *FUSCA3* (*FUS3*), and *LEAFY COTYLEDON2* (*LEC2*) [10]. *LEC2* mainly regulates lipid synthesis, and its target genes are *ABI3*, *FUS3*, *LEC1,* and *WRI1* [11]. For example, the overexpression of *LEC2* in tobacco increased total fatty acid content by 6.8% [12]. Third, among *NFYB* transcription factors, *LEC1* is a subunit encoding CCAAT-Box binding factor *HAP3*, which is a key regulator of embryonic development. Although the overexpression of *ZmLEC1* and *ZmWRI1* in maize can increase the corn oil content by 48%, it also affects the normal growth and development of seeds [13]. Fourth, *ASIL1* (*Arabidopsis 6b-interacting protein 1-like1*) protein could indirectly inhibit *LEC1*, *LEC2*, *FUS2,* and *ABI3*, thus regulating the seed filling process [14].

*WRI1* (*WRINKLED1*) is the master transcription factor found to directly regulate glycolysis and fatty acid synthesis. In *Arabidopsis*, *wri1-1* is a mutant with uneven epidermis and atrophic grain screened from seeds [15]. *WRI1* plays a significant role in regulating seed oil content in the complex regulatory network composed of transcription factors [16]. The phosphorylated structure in *WRI1* is called PEST-motif. The transcription of PEST-motif can increase the content of triacylglycerol in seeds, and the deletion or mutation of this motif can increase the stability of *WRI1* proteins [17]. Moreover, in the early stage of seed development, *WRI1* promotes the high expression of genes related to triglyceride synthesis by regulating a variety of genes involved in fatty acid synthesis, glycolysis, and biotin synthesis [18]. Overexpression of the *WRI1* gene during seed development could increase the oil content in seeds and other organs [19]. For example, the overexpression of *BnWRI1* in *Brassica napus* could promote the expression of key enzymes in the glycolysis pathway and in fatty acid synthesis, which confirms its ability to enhance the accumulation of oil in plant seeds and leaves [20]. In maize, *WRI1* is mainly expressed in the embryo and endosperm of seeds, promoting the increase of fatty acid content in mature maize grains, and does not affect its seeding growth, starch content, or grain yield [13]. Moreover, the expression of the *WRI1* gene in the middle and late stages of seed development can promote the accumulation of seed oil and inhibit the accumulation of seed protein, which indicates that *WRI1* has a certain role in maintaining the balance of seed oil and protein [21]. *P. ostii* was used in this study, and the key transcription factor gene *PoWRI1* was cloned. After transformed into *Arabidopsis*, the phenotypic observation, gene expression of the transgenic *Arabidopsis* seeds, and lipid assay were analyzed in order to study the regulation function of transcription factor *PoWRI1* on seed lipid accumulation. Our research lays the foundation for further identification of the *PoWRI1* gene and its application in molecular genetic improvement of oil composition.

## 2. Results

### 2.1. Isolation and Sequence Analysis of PoWRI1 from Paeonia ostii

**Figure 1:**
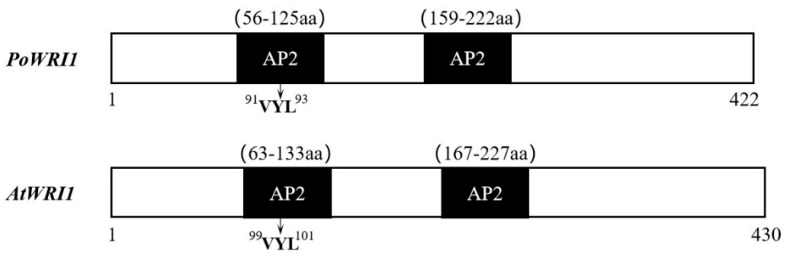
The structural characteristics of *PoWRI1* (MW930196) and *AtWRI1* (NP_001030857.1).

**Figure 2:**
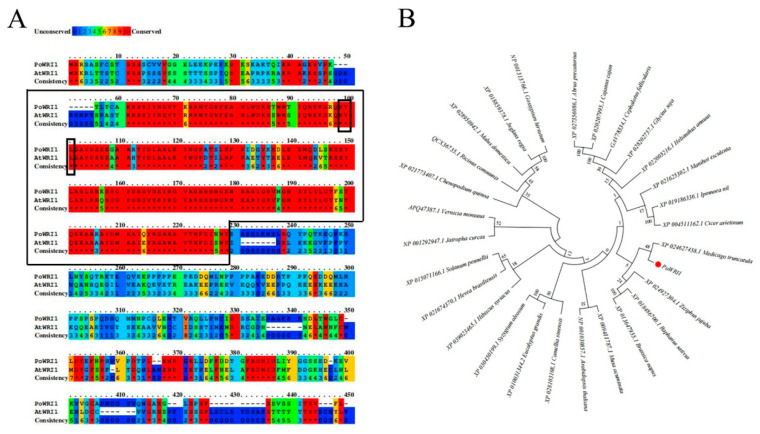
Sequence analysis of *PoWRI1*. (**A**) Alignment of protein sequence of *PoWRI1* (MW930196) and *AtWRI1* (NP_001030857.1). Most of the similarity between protein sequences of *PoWRI1* and *AtWRI1* occurs at the AP2 regions of the protein (highlighted by boxes). Conservation of amino acids is denoted by different colors as illustrated by the scale bar. (**B**) Phylogeny tree of *WRI1* homologs including *Paeonia ostii* (MW930196), *Vernicia montana* (APQ47387.1), *Cephalotus follicularis* (GAV78334.1), *Arabidopsis thaliana* (NP_001030857.1), *Jatropha curcas* (NP_001292947.1), *Gossypium hirsutum* (NP_001313766.1), *Ricinus communis* (QCX36735.1), *Cicer arietinum* (XP_004511162.1), *Musa acuminata* (XP_009411787.1), *Eucalyptus grandis* (XP_010031344.2), *Brassica napus* (XP_013647955.1), *Solanum pennellii* (XP_015071166.1), *Raphanus sativus* (XP_018486700.1), *Juglans regia* (XP_018859378.1), *Ipomoea nil* (XP_019186336.1), *Cajanus cajan* (XP_020207995.1), *Manihot esculenta* (XP_021625302.1), *Hevea brasiliensis* (XP_021674370.1), *Chenopodium quinoa* (XP_021773407.1), *Helianthus annuus* (XP_022005216.1), *Medicago truncatula* (XP_024627458.1), *Ziziphus jujuba* (XP_024927304.1), *Abrus precatorius* (XP_027356086.1), *Camellia sinensis* (XP_028103108.1), *Glycine soja* (XP_028202757.1), *Malus domestica* (XP_028950942.1), *Syzygium oleosum* (XP_030450199.1) and *Hibiscus syriacus* (XP_039023465.1).

Specific primers were designed according to the full-length cDNA sequence of the *WRI1* gene from the transcriptome database (PRJNA317164) [22] (Table S1) and employed to clone the gene named *PoWRI1*. The total length of the *PoWRI1* gene is 1413 bp, which has a complete open reading frame of 1269 bp and encodes 422 amino acids (The accession number is MW930196). After being analyzed by the ProtParam online tool, the isoelectric point of *PoWRI1* is 5.68 and the molecular weight of *PoWRI1* was 47.0 kDa. It is worth noting that the instability coefficient of PoWRI1 protein was 52.41, so PoWRI1 belongs to the unstable protein. *PoWRI1* has two conserved AP2 domains, which are located between amino acids 56 and 125 and between amino acids 159 and 222. The Motif “VYL” encoded by a 9-bp exon is between amino acids 91 and 93, which is similar to the structure of *Arabidopsis thaliana* (Figure 1). Except the regions of C-terminal and N-terminal, the results of sequences alignment analysis showed that the similarity of the *PoWRI1* and *WRI1* protein sequence of *Arabidopsis thaliana* seems high. The regions of the two AP2 conserved domains between PoWRI1 and AtWRI1 protein shared especially high similarity (Figure 2A). In addition, the phylogenetic tree was constructed with the neighbor-joining method by MEGA 7.0 following multiple alignments of protein sequences (Figure 2B). The alignment results showed that the WRI1 protein sequence has a consistency in different plants and PoWRI1 protein has the highest evolutionary similarity with *Medicago truncatula* (XP_024627458.1).

### 2.2. Expression Level Analysis of PoWRI1 in P. ostii

**Figure 3:**
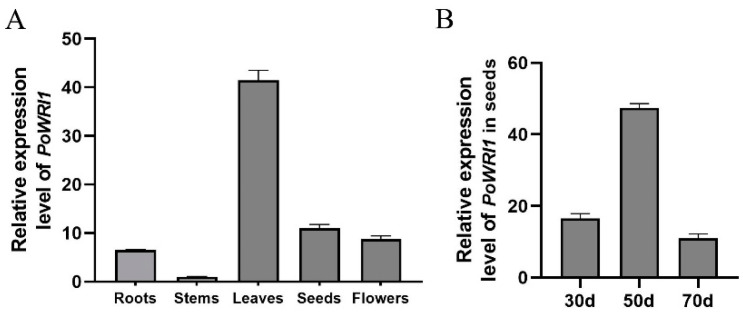
Expression level analysis of *PoWRI1* in *P. ostii*. (**A**) Relative expression level of *PoWRI1* of different tissues in *P. ostii*. The reference gene was *Ubiquitin* (JN699053), the primers were based on the coding sequences of *PoWRI1* (MW930196). Total RNA was extracted from roots, stems, leaves, flowers, and seeds (70d) of *P. ostii*. Values are the means ± SE of three replicates carried out on cDNAs obtained from three independent mRNA extractions. (**B**) Relative expression level of *PoWRI1* in seeds of different stages. The reference gene was *Ubiquitin* (JN699053), the primers were based on the coding sequences of *PoWRI1* (MW930196). Total RNA was extracted from the seeds of 30d, 50d, and 70d. Values are the means ± SE of three replicates carried out on cDNAs obtained from three independent mRNA extractions.

In order to detect the expression of *PoWRI1* for different tissues in *P. ostii*, qRT–PCR was used to analyze the roots, stems, leaves, seeds, and flowers in *P. ostii*. *Ubiquitin* (JN699053) was used as the reference gene. As shown in Figure 3A, the expression level of *PoWRI1* in different tissues was different, and showed a higher expression level in leaves, followed by seeds, compared with other tissues.

We further investigated the expression level of *PoWRI1* in *P. ostii* seeds at 30d (i.e., days after flowering), 50d, and 70d. qRT–PCR results showed that the expression levels of *P. ostii* seeds in 50d were higher than the other two stages (Figure 3B). With the development of seeds in *P. ostii*, the *PoWRI1* gene was upregulated and then downregulated.

### 2.3. Subcellular Localization of the PoWRI1 Protein

**Figure 4:**
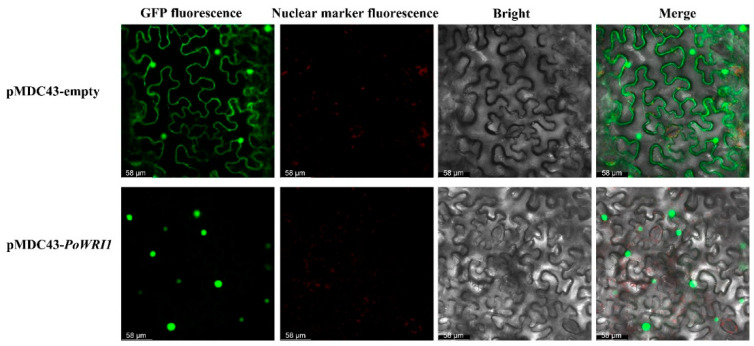
Subcellular localization of the PoWRI1 protein. An empty vector pMDC43 and pMDC43-*PoWRI1* transiently expressed in *N. benthamiana* leaves. Bars = 58 μm.

To further determine the subcellular localization of the PoWRI1 protein, the expression vector pMDC43-*PoWRI1* was constructed and transformed into *N. benthamian* by the Fast Agro-mediated Seedling Transformation (FAST) method [23] using an empty vector pMDC43 as a control. As is shown in Figure 4, the GFP green fluorescence sites were widely distributed in the whole plasma membrane system and nucleus in the pMDC43 empty vector. However, the GFP green fluorescence sites of pMDC43-*PoWRI1* were located at the nucleus, indicating that *PoWRI1* was a nuclear localization protein and consistent with the subcellular localization prediction result.

### 2.4. Genetic Transformation of PoWRI1 Gene in A. thaliana

After pCAMBIA1301-*PoWRI1* overexpression, the vector was constructed and transformed into Agrobacterium tumefaciens strain EHA105 by a freeze–thaw method. The inflorescence of wild-type *Arabidopsis thaliana* was infected. After a series of screening and culture, T3 generation seeds were harvested.

**Figure 5:**
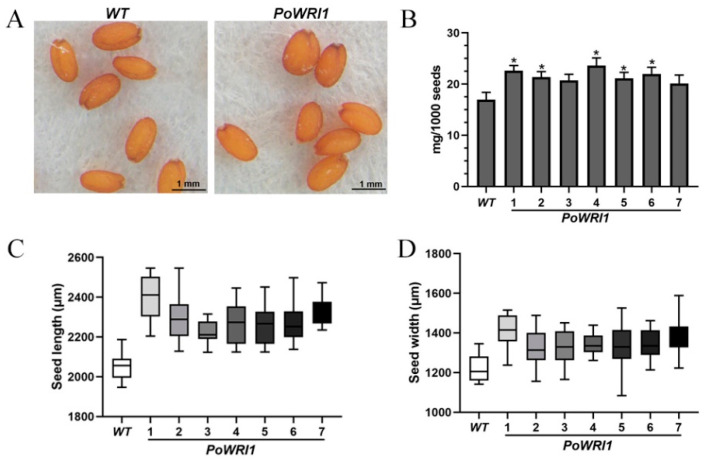
Phenotypic observation of transgenic *Arabidopsis*. (**A**) Dry seeds of *WT* and *PoWRI1* observed under a stereomicroscope. Bars indicate 1 mm. (**B**) 1000 dry seeds weigh of *WT* and *PoWRI1* (Line 1–7). Error bars are s.e. (*n* = 3). * indicates significant differences between *WT* and *PoWRI1* (Line 1–7) (*p* < 0.05). (**C**) Seed length of *WT* and *PoWRI1* (Line 1–7). Error bars are s.e. (*n* = 23). (**D**) Seed width of *WT* and *PoWRI1* (Line 1–7). Error bars are s.e. (*n* = 23).

The seeds of T3 generation *Arabidopsis thaliana* were collected after drying in the oven. The length and width of seven transgenic lines seeds and wild-type *Arabidopsis thaliana* seeds were measured. As shown in Figure 5A, compared with wild-type ones, the seeds of transgenic *Arabidopsis* are plumper and larger in shape and darker in color. In addition, the dried *Arabidopsis* seeds were randomly selected to calculate their average 1000 seed weight and standard deviation. The 1000 seed weight of transgenic *Arabidopsis* plants significantly higher compared with that of wild-type *Arabidopsis* (Figure 5B). The average weight of each wild-type seed was 16.97 μg and the average weight of each transgenic seed was 21.64 μg, increased by 27.5% compared with wild-type plants. The length and width of T3 generation seeds of 7 transgenic *Arabidopsis* plants were significantly larger than those of wild-type ones (Figure 5C,D).

### 2.5. Expression Level Analysis of Transgenic A. thaliana

**Figure 6:**
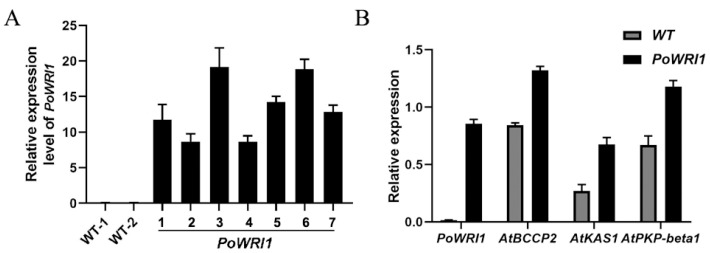
Expression level analysis of transgenic *A. thaliana*. (**A**) Relative expression level of *PoWRI1*. *WT-1* and *WT-2* is wild-type lines, 1–7 are *PoWRI1* transgenic lines. The reference gene was *AtActin* (AK230311.1), the primers were based on the coding sequences of *PoWRI1* (MW930196). Total RNA was extracted from *Arabidopsis* leaves. Values are the means ± SE of three replicates carried out on cDNAs obtained from three independent mRNA extractions. (**B**) Relative expression level of *WRI1* and downstream target genes in *Arabidopsis* leaves. The reference gene was *AtActin* (AK230311.1), the primers were based on the coding sequences of *PoWRI1* (MW930196), *AtBCCP2* (AT5G15530), *AtKAS1* (AT5G46290) and *AtPKP-β1* (AT5G52920). Total RNA was extracted from *Arabidopsis* leaves. Values are the means ± SE of three replicates carried out on cDNAs obtained from three independent mRNA extractions.

In order to detect the expression of the target gene *PoWRI1* in transgenic *Arabidopsis*, qRT–PCR was used to analyze the transgenic *Arabidopsis* plants. Taking two wild-type *Arabidopsis* plants as controls, seven transgenic *Arabidopsis* plants were selected. As shown in Figure 6A, the expression level of *PoWRI1* in different transgenic *Arabidopsis* plants was different, and showed a higher expression level compared with the control.

To further investigate the function of *PoWRI1* in gene expression regulation, specific primers were used to detect the expression level of downstream target genes of *WRI1*, including *biotin carboxyl carrier protein isoform 2* (*BCCP2*), *3-ketoacyl-acyl carrier protein synthase 1* (*KAS1*) and *plastid pyruvate kinase beta subunit 1* (*PKP-β1*). qRT–PCR results showed that the expression levels of these genes in transgenic *Arabidopsis* were higher than those in wild-type *Arabidopsis* (Figure 6B). The expression of *BCCP2* in transgenic *Arabidopsis* was the highest (56% higher than that in wild-type ones), the expression of *KAS1* was 76% higher than that of wild-type ones, and the expression of *PKP-β1* was 60% higher than that of wild-type ones. The results showed that the overexpression of *PoWRI1* in *Arabidopsis* increased the expression of downstream genes related to lipid synthesis.

### 2.6. High Fatty Acid Content in Transgenic Arabidopsis Seeds

**Figure 7:**
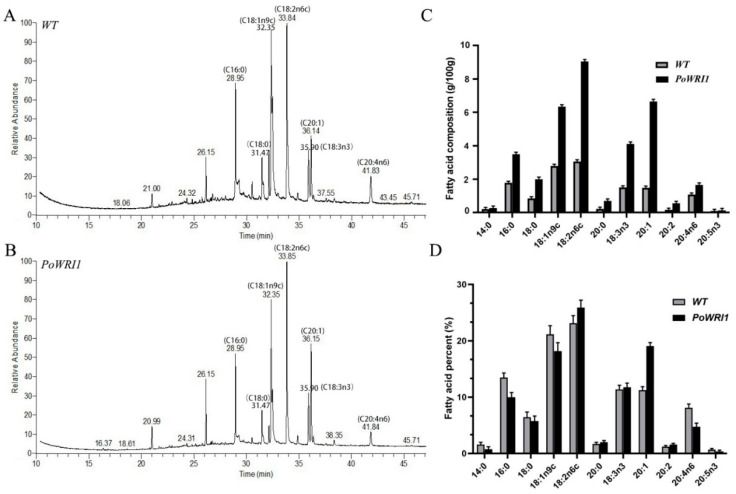
Analysis of fatty acid content in *Arabidopsis* seeds. (**A**,**B**) GC-MS analysis of fatty acids isolated from the *Arabidopsis* seeds. (**C**,**D**) Fatty acid content and percentages of each component isolated from the *Arabidopsis* seeds. Error bars are s.e. (*n* = 3).

To identify the function of the *PoWRI1* gene in fatty acid biosynthesis and oil accumulation, we verified whether the overexpression of the *PoWRI1* gene leads to the increase of total fatty acid content in *Arabidopsis* seeds. Fatty acids in the seeds of transgenic lines (1, 4, 6) and wild-type lines were detected and analyzed by Thermo Trace1310 ISQ GC-MS with nonadecanoic acid (C19:0) as an internal standard. A total of 11 fatty acid were detected in both transgenic and wild-type *Arabidopsis* (Figure 7A,B). The peak area of the measured results was analyzed by GC-MS and the fatty acid content of *Arabidopsis* seeds was calculated. As shown in Figure 7C, the content of each fatty acid in transgenic plants increased compared with that in wild type. Notably, the content of long-chain fatty acids and unsaturated fatty acids increased significantly. Among them, oleic acid (C18:1n9c), linoleic acid (C18:2n6c), α-linolenic acid (C18:3n3), and arachidonic acid (C20:0) increased nearly two times compared with wild-type ones. The contents of palmitic acid (C16:0) and stearic acid (C18:0) were nearly doubled compared with wild-type ones. The proportion of each fatty acid component in the seeds of transgenic and wild-type plants was also significantly different (Figure 7D). The proportion of unsaturated fatty acids in transgenic *Arabidopsis* seeds was higher than that in wild-type ones.

## 3. Discussion

### 3.1. Structural Characteristics of PoWRI1 and Its Expression Levels in P. ostii

*WRI1* was first identified in *Arabidopsis* [4], and its orthologs have been identified from many plants such as *Cocos nucifera* [24], *Brassica napus* [20], *Ricinus connunis* [25], *Glycine max* [26], *Avena sativa* [27], and *Zea mays* [28]. The full-length cDNA sequence of the *PoWRI1* gene of *P. ostii* was also identified and cloned in our study. It was confirmed that *PoWRI1* belongs to the *AP2/EREBP* transcription factor gene family. In the same way as *AtWRI1*, *PoWRI1* has a PEST motif despite the divergence between its C-terminal sequences (Figure S1), suggesting that this region is conserved and contributes to *WRI1* stability [17]. Although the C-terminal regions of *AtWRI1* and *PoWRI1* are strikingly diverged, bioinformatics analysis showed that *PoWRI1* and *AtWRI1* shared a 93% sequence similarity in the AP2 domain and a 94% sequence similarity in the second AP2 domain. The differences in these individual amino acids of the AP2 domains may be due to species differences. In addition, studies have shown that there is an important amino acid “VYL”, which is conserved in a number of *WRI1* orthologs discovered in many plant species [29]. The mutation of a single amino acid in “VYL” can lead to the impairment of the function of *WRI1* protein in *Arabidopsis* [30]. In this study, the PoWRI1 protein in the first AP2 domain has the same amino acid “VYL” as the WRI1 sequence of *Arabidopsis*. It can be inferred that the *PoWRI1* of *P. ostii* has the essential role of “VYL” for *PoWRI1* function.

During the period of seed development and filling, photosynthetic carbons are partitioned into starch, oil and proteins, and other different storage compounds, which are highly regulated by *LEAFY COTYLEDON 1* (*LEC1*), *LEC2,* and *WRI1* [31]. In *P. ostii*, the development of seeds can be divided into three stages. The level of fatty acid is relatively low in the first stage. Then there is a period of rapid oil accumulation. Finally, there is a decrease with the seed approaching full maturity [32]. The expression level of *PoWRI1* was first upregulated and then downregulated with the development of seeds, which was consistent with the trend of oil accumulation.

### 3.2. Phenotype of Transgenic Arabidopsis Thaliana and Overexpression of WRI1 Downstream Gene

It is known that the enzymes involved in sugar metabolism and hexose and sucrose accumulation affect the number and size of seed cells during seed development, which in turn affects the quality and size of seeds [33,34]. In addition, as the central regulator of the fatty acid biosynthesis pathway, the *WRI1* transcription factor can specifically and positively involved glycolysis and fatty acid biosynthesis [35] through affecting the expression of related genes in glycolysis pathways to regulate key enzymes involved in the process, finally affecting the phenotype of *Arabidopsis* seeds of glycometabolism. After the preliminary phenotypic observation of transgenic *Arabidopsis* seeds, it was found that the size and weight of transgenic *Arabidopsis* seeds were significantly higher than that of wild-type ones, indicating that the *PoWRI1* gene has a certain role in promoting the growth and development of plant seeds. Meanwhile, it can be found that the expression levels of target genes of *WRI1* downstream including *BCCP2*, *KAS1,* and *PKP-β1* in transgenic *Arabidopsis* were higher than those in wild-type *Arabidopsis*, which were similar to the results that overexpression of *RcWRI1* significantly upregulated the expression of *pyruvate kinase alpha subunit* (*PKP-α*), *acyl carrier protein 1* (*ACP1*), *pyruvate dehydrogenase E1 component alpha subunit* (*PDH-Elα*), *BCCP2*, *KAS1,* and *PKP-β1* in *N. benthamiana* leaves [25], regulating the expression of the above essential genes in FA biosynthesis [36,37,38,39]. As the target genes of *WRI1*, these genes are involved in glycolysis and fatty acid synthesis. The promoter region of *WRI1* transcription factor’s downstream gene has a highly conserved sequence, which is called AW-box (CnTnG(n)7CG) or 15 bp element (CAAAAG(T/G)AGG(G/A)APTT). *WR11* regulates downstream gene transcription by binding AW (ASML1/WRI1)-box, which plays an important role in regulating the carbon flow from sugar to oil during seed development [35,40].

### 3.3. Significant Increase of Fatty Acid Content in Transgenic Arabidopsis Thaliana

In fact, *WRI1* affects oil accumulation by regulating the metabolic process during seed development, especially the glycolysis pathway [41,42]. Glycolysis provides raw materials for fatty acid synthesis. The overexpression of *WRI1* regulates lipid metabolism by up regulating the expression of target genes involved in glycolysis and fatty acid synthesis, thereby promoting the carbon flow from glycolysis to fatty acid synthesis in seeds and improving the content of triacylglycerol in seeds and seedlings [26]. Research has found that all of the *WRI1* homologs also induced oil accumulation in *N. benthamiana*. The increase of unsaturated fatty acids was not obvious [27]. Moreover, the transcription levels of fatty acid synthetase, plastid transporter and key enzymes of plastid glucose metabolism in oil palm with high oil content were enhanced compared with those in date palm. The expression level of the *WRI1* transcription factor homologous transcript in oil palm was 57 times higher than that in date palm, while the expression level of enzymes related to triglyceride assembly was similar in both oil palm and date palm. These results further indicated that *WRI1* promoted lipid synthesis by regulating plastid pyruvate supply and fatty acid synthesis, but was not directly involved in fatty acid modification and triglyceride synthesis [43]. Our results showed that the content of fatty acids in the seeds of transgenic *Arabidopsis* was more than twice as high as that of wild-type ones. Therefore, the *WRI1* gene significantly promoted the increase of fatty acid content in transgenic *Arabidopsis* seeds. It’s worth noting that the content of unsaturated fatty acids and long chain fatty acids in transgenic *Arabidopsis* were also higher than those of wild-type ones. In other words, *PoWRI1* has a significant effect on the increase of unsaturated fatty acids, suggesting that *PoWRI1* helps the synthesis of an essential enzyme promoting the dehydrogenation of saturated fatty acids to unsaturated fatty acids, such as Fatty acid desaturase (FAD) [44].

Currently, the demand for vegetable oil is increasing. Improving oil content and fatty acid composition of oil crops to meet high demand of vegetable oil market represents a promising strategy, and the heterologous overexpression of *WRI1* is an effective measure. For example, transferring the *BnWRI1* gene into *Arabidopsis* will increase the oil content, seed volume, and weight of transgenic *Arabidopsis* seeds [20], which is similar to the results of this experiment. As a result, our study provides insights into the transcriptional activation of glycolysis and fatty acid biosynthesis pathways in *Arabidopsis*, and lays a foundation for further elucidating the regulatory network controlling seed oil accumulation.

## 4. Materials and Methods

### 4.1. Plant Materials

The seeds (30d, 50d, and 70d), roots, stems, leaves, and flowers of 3-year-old *P. ostii* from the germplasm repository of the Horticulture and Plant Protection College, Yangzhou University, Jiangsu Province, P.R. China (32°23′31″ N, 119°24′50″ E) were collected and stored in liquid nitrogen. They were stored in −80 °C for RNA extraction.

The transformed plant material is the seed of *Arabidopsis thaliana* of Columbia *Col-0* type and stored at 4 °C (21 days after anthesis).

### 4.2. Gene Cloning and Sequence Analysis

After the seeds grinding into powder, RNA was separately extracted using the Mini BEST Plant RNA Extraction Kit (TaKaRa, Tokyo, Japan), and all RNA samples were checked using Nanodrop 2000C (Thermo Scientific). According to PrimeScript^®^ RT reagent Kit with gDNA eraser (Perfect Real Time) (TaKaRa, Tokyo, Japan), RNA was reserved into cDNA. The specific primers *PoWRI1*-F and *PoWRI1*-R were designed by Primer 5.0 software for the PCR amplification (Table S1). The PCR reaction was as follows: 1 cycle of 94 °C for 5 min; 30 cycles of 94 °C for 30 s, 55 °C for 30 s, 72 °C for 2 min; and one cycle of 72 °C for 10 min. After testing by 1% (*w*/*v*) agarose gel electrophoresis, the *PoWRI1* PCR products were cloned into the pClone007 Vector and sequenced.

Protparam (http://web.expasy.org/protparam/) (accessed on 30 April 2021) was used to analyze the amino acid composition, relative molecular weight, isoelectric point, and other physical and chemical properties. The conserved domain of PoWRI1 protein was analyzed by CD-Search tool of the National Center for Biotechnology Information site (NCBI, https://www.ncbi.nlm.nih.gov) (accessed on 30 April 2021). The protein sequence of *AtWRI1* (NP_001030857.1) was obtained from NCBI and the alignment was analyzed by the PRALINE program (http://www.ibi.vu.nl/programs/pralinewww/) (accessed on 30 April 2021). A Neighbor-Joining phylogenetic tree was generated with MEGA 7.0, and bootstrap values were set as 1000 bootstrap replicates [45].

### 4.3. Construction of Expression Vector

The specific primers attB-*PoWRI1*-F and attB-*PoWRI1*-R were designed (Table S1). The PCR products were then cloned into the Invitrogen GATEWAY^TM^ pDONR/Zeo vector (Thermo Fisher Scientific, Waltham, MA, USA) using the BP reaction and sub-cloned (LR reaction) into the plant GATEWAY™ binary vector: pMDC43 for transient expression in *N. benthamiana* to determine the subcellular localization of the PoWRI1 protein.

Based on the obtained full-length sequence of *PoWRI1*, combined with the restriction site of the binary expression vector pCAMBIA1301, the recombinant plant transgenic vector was constructed by T4 DNA ligase Buffer. The *Bsa I* restriction sites on the polyclonal site of pCAMBIA1301 was used to cleave the vector. At the same time, the coding region of *PoWRI1* gene was amplified with primers *PoWRI1*-*Bsa I*-F/R (including the corresponding *Bsa I* restriction sites) (Table S1). The recombinant plasmid pCAMBIA1301-*PoWRI1* was constructed by ligating the target fragment to the vector with T4 DNA ligase Buffer.

### 4.4. Overexpressing PoWRI1 in the Arabidopsis

The expression vector pCAMBIA1301-*PoWRI1* plasmids were used for the transformation of competent cells of *Agrobacterium tumefaciens* strain EHA105. *Arabidopsis Col-0* plants were transformed using the floral-dip method [46]. The mature seeds were collected and recorded as T0. The seeds of T1 generation were screened by MS medium containing hygromycin (25 mg/L). When the seedlings grew four rosette leaves, the better growing ones were transplanted into sterilized soil (nutrient soil: perlite: vermiculite = 2:1:1) and cultured at 22 °C for 14 h in light (100–150 μmol m^−2^ s^−1^ illumination), 18 °C for 10 h in dark, and the humidity was 70–80%. The seeds were collected and recorded as T2 generation after flowering and fruiting. T2 generation seeds were screened with hygromycin (25 mg/L) until all of them were resistant seedlings, and then were screened and cultured according to the above method until T3 generation seeds were harvested and transgenic plants were identified by PCR. 1–7 transgenic lines were in good growing condition and were selected from the T3 generation seed.

### 4.5. Phenotypic Observation of Transgenic Arabidopsis

After the flowering and pod ripening of *Arabidopsis thaliana*, pods were harvested before the coat was split and placed in the oven. After drying at 37 °C for 24 h, the coat of the pod was removed and the seeds of T3 generation *Arabidopsis thaliana* were collected. The length and width of seeds of different transgenic and wild-type *Arabidopsis* were measured. Transgenic and wild-type *Arabidopsis* randomly measured 30 seeds, repeated three times, using SAS/STAT 6.12 statistical analysis software for variance analysis of the results, using GraphPad Prism 8.0.2 software to draw pictures about the length and width of seeds.

1000 dry seeds of transgenic and wild-type *Arabidopsis* were weighed randomly and repeated for three times. The average seed weight and standard deviation were calculated and plotted by GraphPad Prism 8.0.2 software.

### 4.6. Quantitative Real-Time PCR Analysis

The total RNA of different tissues and seeds at different stages of *P. ostii* was extracted, according to the Mini BEST Plant RNA Extraction Kit (TaKaRa, Tokyo, Japan). Then, the RNA was reverse-transcribed into cDNA by the superscript first-strand synthesis system (Prime-Script^®^ RT Reagent Kit With gDNA Eraser, TaKaRa, Tokyo, Japan). Using *Ubiquitin* (JN699053) as an internal reference, real-time quantitative PCR (qRT-PCR) was introduced to analyze the expression levels of *PoWRI1* in different tissues and seeds at different stages of *P. ostii* with a BIO-RAD CFX Connect Optics Module (Bio-Rad, Des Plaines, IL, USA).

The total RNA of T3 generation transgenic and wild-type *Arabidopsis* leaves in full bloom was extracted by above mentioned method. Using the *Actin* (AK230311.1) gene as an internal reference, real-time quantitative PCR (qRT–PCR) was introduced to analyze not only the expression levels of *PoWRI1*, but also the expression levels of downstream target genes (*AtBCCP2*, *AtKAS1* and *AtPKP-β1*) of *WRI1* in transgenic and wild-type *Arabidopsis*.

Specific primers were designed using Primer 5.0 (Table S1). The formula 2^−ΔΔCt^ was referred to calculated their values. A final volume of 25 μL (12.5 μL 2 × SYBR Premix Ex Taq, 2 μL cDNA solution, 2 μL mix solution of primers and 8.5 μL ddH_2_O) was the system to peform qRT-PCR. The reaction conditions were 95 °C for 30 s, 40 cycles at 95 °C for 5 s, 52 °C for 30 s, and 72 °C for 30 s.

### 4.7. Analysis of Fatty Acids Content in Seeds of Transgenic Arabidopsis

Firstly, the dry wild-type and T3 generation transgenic *Arabidopsis* seeds, several zeolites, pyrogallic acid, 95% ethanol, and hydrochloric acid solution were added into the flask and mix well. After hydrolysis in water bath and cooling to room temperature, 95% ethanol was added to the hydrolysate. The mixture was then shaken and collected in a constant weight flask. After evaporating and drying in an oven, 2% sodium hydroxide methanol and 14% boron trifluoride methanol were added to the oil extract respectively. Then, n-hexane was added for centrifugation and stratification. The supernatant and n-hexane were dissolved again, and the volume was then fix to 1 mL and mixed well. Finally, after filtration, the membrane was tested. Trace1310 ISQ gas chromatography-mass spectrometry (GC-MS) of Thermo company (Germany) was used to detect fatty acids. The chromatographic column was TG-5MS (30 m × 0.25 mm × 0.25 μm).

## 5. Conclusions

The *PoWRI1* gene, which is located at the nucleus and is associated with the oil accumulation process, was identified and cloned. The overexpression of *PoWRI1* in *Arabidopsis* increased the expression level of downstream target genes such as *BCCP2*, *KAS1,* and *PKP-β1*. Meanwhile, the seeds of transgenic plants became larger, the oil content increased significantly, and the unsaturated fatty acid content increased, which provides a scientific theoretical basis for the subsequent use of genetic engineering methods to improve the fatty acid composition and content of plant seeds.

## Data Availability

Data are contained within the article or Supplementary Materials.

## Supplementary Materials

The following are available online at https://www.mdpi.com/article/10.3390/ijms22136996/s1. Figure S1: PEST motif analysis of *AtWRI1* and *PoWRI1*; Table S1: Primers used for PCR and qRT–PCR.
