# Supplementary material for: Analysis and Functional Verification of PoWRI1 Gene Associated with Oil Accumulation Process in Paeonia ostii
Source: Int J Mol Sci. 2021 Jun 29;22(13):6996. doi: 10.3390/ijms22136996 (PMC8267616; doi:10.3390/ijms22136996)
Supplement: Supplementary file 1 [file ijms-22-06996-s001.zip › Figure S1.pdf]

>PoWRI1
MKRSASFCSTSSSSCVVVGGELEEKPKPKSKSKAKTQIKRAGKSVPKTLTCARRSSIYRG
VTKHRWTGRFEAHLWDKSTWNTIQNKKGRQVYLGAYDSEEGAARTYDLAALKYWG
PATSLNFPIDGYKKDLEEMQDLSKEEYLASLRRKSSGFSRGVSKYRGVARHHHNGRWE
ARIGRVMGNKYLYLGTFSTQEEAARAYDMAAIQYRGANAVTNFDINNYSASGLKNSL
RQYPQTKEQPKHLNYSQTRKTEQVEEPPPPPEPEDDQMLNPPPPAPEDDHTPPPQEDD
QMLNPPSPSPQDHQMMNPCQLENTVRQLLPNCIDSSAIEPAGFSEHDLTWGLCLDTEF
NPHPVPDTPLENPGELLDFFDDTGFEDNIDLIYGGSSEDKEVKVVGCADNDGSVQNG
AEGLSPSPSSVSSITSVFL
>AtWRI1
MKKRLTTSTCSSSPSSSVSSSTTTSSPIQSEAPRPKRAKRAKKSSPSGDKSHNPTSPASTRR
SSIYRGVTRHRWTGRFEAHLWDKSSWNSIQNKKGKQVYLGAYDSEEAAAHTYDLAAL
KYWGPDTILNFPAETYTKELEEMQRVTKEEYLASLRRQSSGFSRGVSKYRGVARHHHN
GRWEARIGRVFGNKYLYLGTYNTQEEAAAAYDMAAIEYRGANAVTNFDISNYIDRLK
KKGVFPFPVNQANHQEGILVEAKQEVETREAKEEPREEVKQQYVEEPPQEEEEKEEEK
AEQQEAEIVGYSEEAAVVNCCIDSSTIMEMDRCGDNNELAWNFCMMDTGFSPFLTDQ
NLANENPIEYPELFNELAFEDNIDFMFDDGKHECLNLENLDCCVVGRESPPSSSSPLSC
LSTDSASSTTTTTTSVSCNYLV

Figure S1. PEST motif analysis of *AtWRI1* and *PoWRI1*. PEST motif analysis was performed by ePESTfind (http://emboss.bioinformatics.nl/cgi-bin/emboss/epestfind). Predicted potential PEST motifs are highlighted in yellow.
