# Supplementary material for: Analysis and Functional Verification of PoWRI1 Gene Associated with Oil Accumulation Process in Paeonia ostii
Source: Int J Mol Sci. 2021 Jun 29;22(13):6996. doi: 10.3390/ijms22136996 (PMC8267616; doi:10.3390/ijms22136996)
Supplement: Supplementary file 1 [file ijms-22-06996-s001.zip › Table_S1.pdf]

Table S1 **Primers used for PCR and qRT-PCR**

**Name**	**Primer (5'-3')**
PoWRI1-F	GTGAGATAGAAAGAGGAGGAAGT
PoWRI1-R	GCCGCAGAGTTTACACGAGCATA
attB-*PoWRI1*-F	GGGGACAAGTTTGTACAAAAAAGCAGGCTCCATGAAGAGGTCAGCTTCA
attB-*PoWRI1*-R	GGGGACCACTTTGTACAAGAAAGCTGGGTCGAAAACTGAAGTAATTGAAGA
PoWRI1-*Bsa I*-F	CAGTGGTCTCACAACATGAAGAGGTCAGCTTCATT
PoWRI1-*Bsa I*-R	CAGTGGTCTCATACACAAGAAAACTGAAGTAATTG

**Gene ID**	**Name**	**Forward Primer (5'-3')**	**Reverse primer (5'-3')**
AK230311.1	*AtActin*	TCTCCCGCTATGTATGTCGC	TAAGGTCACGTCCAGCAAGG
AT5G15530	*AtBCCP2*	GTGGAACTTGAACTAAAGCAGC	AGGAGAAGGAGGAAGAGCC
AT5G46290	*AtKAS1*	CGATGTTGATGCTTACTACG	TCAAGCCTACGCTCATTCT
AT5G52920	*AtPKP-β1*	CCGCTCGTGTTGAGACTGA	TCCTCCTCACTGTCGGCTTC
MW930196	*PoWRI1*	GAGATAGAAAGAGGAGGAAGT	TATGCTCGTGTAAACTCTGCG
JN699053	*Ubiquitin*	GACCTATACCAAGCCGAAG	CGTTCCAGCACCACAATC
